# DNA Methylation Status of the Interspersed Repetitive Sequences for LINE-1, Alu, HERV-E, and HERV-K in Trabeculectomy Specimens from Glaucoma Eyes

**DOI:** 10.1155/2018/9171536

**Published:** 2018-01-31

**Authors:** Sunee Chansangpetch, Sasiprapa Prombhul, Visanee Tantisevi, Pimpayao Sodsai, Anita Manassakorn, Nattiya Hirankarn, Shan C. Lin

**Affiliations:** ^1^Department of Ophthalmology, Faculty of Medicine, Chulalongkorn University and King Chulalongkorn Memorial Hospital, Thai Red Cross Society, Bangkok, Thailand; ^2^Center of Excellence in Immunology and Immune-Mediated Diseases, Department of Microbiology, Faculty of Medicine, Chulalongkorn University, Bangkok, Thailand; ^3^Department of Ophthalmology, University of California - San Francisco, San Francisco, CA, USA

## Abstract

**Background/Aims:**

Epigenetic mechanisms via DNA methylation may be related to glaucoma pathogenesis. This study aimed to determine the global DNA methylation level of the trabeculectomy specimens among patients with different types of glaucoma and normal subjects.

**Methods:**

Trabeculectomy sections from 16 primary open-angle glaucoma (POAG), 12 primary angle-closure glaucoma (PACG), 16 secondary glaucoma patients, and 10 normal controls were assessed for DNA methylation using combined-bisulfite restriction analysis. The percentage of global methylation level of the interspersed repetitive sequences for LINE-1, Alu, HERV-E, and HERV-K were compared between the 4 groups.

**Results:**

There were no significant differences in the methylation for LINE-1 and HERV-E between patients and normal controls. For the Alu marker, the methylation was significantly lower in all types of glaucoma patients compared to controls (POAG 52.19% versus control 52.83%, *p* = 0.021; PACG 51.50% versus control, *p* = 0.005; secondary glaucoma 51.95% versus control, *p* = 0.014), whereas the methylation level of HERV-K was statistically higher in POAG patients compared to controls (POAG 49.22% versus control 48.09%, *p* = 0.017).

**Conclusions:**

The trabeculectomy sections had relative DNA hypomethylation of Alu in all glaucoma subtypes and relative DNA hypermethylation of HERV-K in POAG patients. These methylation changes may lead to the fibrotic phenotype in the trabecular meshwork.

## 1. Introduction

Epigenetics include the chemical reactions that control the genome activities at certain time points and locations within the DNA [1]. These reactions produce a chemical mark on the DNA that can serve as an additional system to control whether the gene will become functional or silent, without modifying the DNA's base sequences. Major epigenetic mechanisms that have been identified are DNA methylation, chromatin remodeling, deployment of noncoding DNA, and histone modification [2–5].

Several studies in complex multifactorial diseases have reported that elucidating the epigenetic mechanisms have helped clarify the understanding of their etiology and disease progression [2]. Stress, diet, behavior, toxins, and other factors can also activate the processes at the epigenomic level [5]. These epigenetic factors may partly explain the clinical variations seen among various multifactorial diseases such as different onset, severity, and progression, beyond simple genetic determination [6]. One of the major processes in epigenetic modification is DNA methylation. Once the heritable methylation patterns in the DNA are disrupted, the chromatin structure and gene expression can be altered and subsequently result in the creation of an aberrant gene expression. Furthermore, there can be pronounced differences in overall and specific methylation levels between different tissue types as well as between normal cells and pathological cells of the same tissue [4, 7]. These tissue-specific characteristics suggest a potential use of methylation changes as a clinical biomarker indicative for disease.

Glaucoma is a progressive optic neuropathy, which is characterized by typical optic disc changes and associated visual function loss [8]. Elevated intraocular pressure (IOP) is the most significant risk factor and is usually a result of impaired aqueous outflow facility due to trabecular meshwork (TM) dysfunction [9]. Glaucoma-associated genes have been discovered but only a small fraction of glaucoma cases are associated with mutations in these genes. The clinical manifestation of onset and severity of glaucoma vary from person to person and even between eyes of the same patient in most cases. It is possible that epigenetic mechanisms that control gene expression may interact with and be related to glaucoma pathogenesis and progression.

At present, there is limited data on epigenetics and glaucoma. A genome-wide methylation analysis conducted in cultured human TM showed that dexamethasone may induce DNA methylation change at some gene promotors [10]. In another study, there was a significant difference in DNA methylation in peripheral mononuclear cells from patients with primary open-angle glaucoma (POAG) compared to the normal controls [11]. These studies suggest that changes in DNA methylation may have a role in glaucoma pathogenesis. However, certain epigenetic mechanisms may be characteristic for specific tissues [12]. Trabeculectomy section or scleral tissue that contains TM may be a better candidate for the epigenetic study, but to the best of our knowledge, there are no studies that have described methylation in excised ocular tissue.

Dysregulation of the DNA methylation processes can occur either as locally, in the promotors of genes, or globally. The total methylcytosine level can be assessed by chromatographic methods to determine the global methylated levels. But there are some limitations to directly quantitate the global outcome so Weisenberger et al. [13] proposed to measure the methylation at interspersed repetitive sequences (IRSs) instead to reflect the global methylcytosine content. Thus, for this study, we assessed the global methylcytosine content by investigating the IRSs of the CpG-rich regions which exist throughout the genome and comprise approximately 45% of the human genome. Our study focused on the 4 major IRSs: long interspersed nuclear elements-1 (LINE-1), Alu element, human endogenous retrovirus-E (HERV-E), and human endogenous retrovirus-K (HERV-K). In brief, there are two major groups of IRSs based on its location: the DNA transposon (2.8% of human genome) and retrotransposon (42.2% of human genome). Long interspersed nuclear elements or LINEs and short interspersed nuclear elements such as ALU are classified as retrotransposon without long terminal repeats (LTR), while human endogenous retrovirus or HERV is a retrotransposon with LTR retroelements [14].

Taken together, our study aimed to investigate the DNA methylation levels for LINE-1, Alu, HERV-E, and HERV-K in scleral tissue from trabeculectomy sections of glaucoma patients and control.

## 2. Materials and Methods

The study followed the tenets of the Declaration of Helsinki and was approved by the Institutional Review Board of the Faculty of Medicine, Chulalongkorn University, Bangkok, Thailand. Informed consent was obtained from each subject before enrollment.

### 2.1. Subjects

Forty-four patients scheduled for trabeculectomy operation were recruited. Glaucoma diagnosis was based on International Society of Geographical and Epidemiological Ophthalmology (ISGEO) guidelines [15]:

1. In category 1, a visual field defect is consistent with glaucomatous optic neuropathy and either a vertical cup-to-disc ratio (C/D) of at least 0.7 (97.5th percentile) or C/D asymmetry between the right and left eyes of at least 0.2 (97.5th percentile).
2. In category 2, visual field results are not definitive or are unattainable due to patient inability to perform an adequate quality test, and optic disc has C/D of at least 0.9 (99.5th percentile) or C/D asymmetry between the right and left eyes of at least 0.3 (99.5th percentile).
3. In category 3, visual field testing and optic disc examination are not possible in the subject; visual acuity is less than 20/400 (for any ophthalmic pathology) and IOP exceeds the 21 mmHg (99.5th percentile for the population).

Inclusion criteria for all patients included (1) age greater than 18 years, (2) uncontrolled IOP on maximally tolerated medications and/or poor compliance with medical therapy, and (3) structural and/or functional deterioration. All glaucoma subjects were classified into the following: (1) POAG—defined as glaucoma patients with open anterior chamber angle by gonioscopy; (2) primary angle-closure glaucoma (PACG)—defined as glaucoma patients with more than 180 degrees iridotrabecular contact or presence of peripheral anterior synechiae by gonioscopy; and (3) secondary glaucoma—defined as glaucoma with an identifiable cause of increased IOP resulting in glaucomatous optic nerve damage. Patients with mixed mechanisms of glaucoma (e.g., POAG with superimposed secondary glaucoma), had other ocular pathology (except for cataract and primary diseases accounting for secondary glaucoma), or had any ocular surgery 6 months prior to the study and patients who were unable to give consent were excluded. Secondary glaucoma patients who had the family history of glaucoma or had any glaucoma suspect sign in the fellow eye were also excluded. All the diagnostic and glaucoma subtype classifications were performed by glaucoma specialists (VT, AM, and SC).

The study was conducted from September to October 2014 at the Ophthalmology Department, King Chulalongkorn Memorial Hospital. Among the 44 recruited patients, 16 had POAG, 12 had PACG, and 16 had secondary glaucoma.

### 2.2. Control Group

Ocular tissue from normal controls were obtained from collaborative partners: the Department of Microbiology, Faculty of Medicine, Chulalongkorn University, and the Department of Forensic Medicine, Police General Hospital, with informed consent from authorized representatives. Subjects included those who had no history of glaucoma according to available medical records and interviews from relatives. To ensure the integrity of specimen quality for optimal measurement of methylation status (which has been shown to be preserved within 48–72 hours postmortem [16, 17]), we collected ten scleral/trabecular tissues from subjects within 24 hours of expiration. The tissue collection procedure was similar to what was done in the glaucoma group, which is described below.

### 2.3. Specimen Collection

The tissue was collected from the sclerostomy during the performance of a standard trabeculectomy, where a section of half-thickness scleral tissue containing TM is cut (block-shaped) to make a connection between the anterior chamber and the subconjunctival space. These trabeculectomy sections, which were approximately 1 mm in width and 1 mm in length, were immediately transported in liquid nitrogen to the laboratory unit for DNA extraction processing and global methylation analysis.

### 2.4. DNA Preparation for Combined-Bisulfite Restriction Analysis (COBRA)

DNA from samples was isolated using QIAamp DNA Micro Kit (QIAGEN, Valencia, CA, USA). DNA quantification was measured by using a NanoDrop 2000c Spectrophotometer (Thermo Fisher Scientific, Waltham, MA, USA). Since the sclera is a fibrillar collagen-type tissue with minimal DNA content, our study used 20 *μ*L of DNA elutes in the bisulfite conversion experiment using EZ DNA Methylation-Gold Kit (Zymo Research, Orange, CA, USA), according to the manufacturer's recommended protocol. One to two microliters of bisulfite-converted DNA were used as a template for the COBRA PCR as a quantitative method to study the DNA methylation of each repetitive sequence. Bisulfite-treated DNA from HeLa, Daudi, and Jurkat have been used as positive controls for each PCR reaction. Specific primers for repetitive sequence markers (i.e., LINE-1, Alu, HERV-E, and HERV-K) were selected and mixed into the PCR reactions. Protocols for COBRA PCR and band measurements have been previously described [14, 18–20]. Polyacrylamide gel electrophoresis revealed banding patterns after restriction enzyme digestion. This COBRA PCR technique is a standard combined-bisulfite method for detecting the methylation of the CpG loci by using a specific set of conserved primers for each repetitive sequence.

### 2.5. Methylation Analyses of Each Repetitive Marker

Percentage of global methylation level has been determined to demonstrate an overall methylation level for each target. Formulas and equations for methylation calculations have been previously described [14]. Results from methylation profiles for LINE-1 and Alu markers are categorized into 4 groups according to the prevalence of methylated/unmethylated CpG occurrence at each specific position: % ^m^C^m^C, % ^m^C^u^C, % ^u^C^m^C, and % ^u^C^u^C.

For LINE-1, numbers of CpG dinucleotides of each motif were normalized and the measured band intensity were divided according to the sizes to generate parameters as follows: %92 bp/92 = *A*, %60 bp/56 = *B*, %50 bp/48 = *C*, %42 bp/40 = *D*, %32 bp/28 = *E*, and [(*D* + *E*) − (*B* + *C*)]/2 = *F*. Percentage of global methylation was calculated by the following formula: [(*A* + 2*C* + *F*) × 100]/(2*A* + 2*B* + 2*C* + 2*F*) = % global methylation. Percentage of hypermethylation pattern (% ^m^C^m^C) at both CpG motifs was calculated by [(*C*/2) × 100]/[(*C*/2) + *A* + *B* + *F*] = % ^m^C^m^C. Percentages of partial methylation of ^m^C^u^C and ^u^C^m^C were calculated by (*A* × 100)/[(*C*/2) + *A* + *B* + *F*] = % ^m^C^u^C and (*F* × 100)/[(*C*/2) + *A* + *B* + *F*] = % ^u^C^m^C, respectively. Percentage of hypomethylation (% ^u^C^u^C) was calculated by (*B* × 100)/[(*C*/2) + *A* + *B* + *F*] = % ^u^C^u^C.

For the Alu marker, we generated parameters to be used for normalization and calculation of the methylation level of each motif as follows: %133 bp/133 = *A*, %58 bp/58 = *B*, %75 bp/75 = *C*, %90 bp/90 = *D*, %43 bp/43 = *E*, and [(*E* + *B*) − (*C* + *D*)]/2 = *F*.

Methylation profiles for the Alu marker have been measured and calculated by the following formulas: global methylation level (% ^m^C) = [100 × (2*F* + *D* + *C*)]/(2*A* + 2*C* + 2*D* + 2*F*), hypermethylation pattern (% ^m^C^m^C) = (100 × *F*)/(*A* + *C* + *D* + *F*), partial methylation (% ^m^C^u^C) = (100 × *D*)/(*A* + *C* + *D* + *F*), partial methylation (% ^u^C^m^C) = (100 × *C*)/(*A* + *C* + *D* + *F*), and hypomethylation (% ^u^C^u^C) = (100 × *A*)/(*A* + *C* + *D* + *F*). As for the HERV methylation levels, the summation of the methylated motifs was calculated by using either the percentage of the band intensity measurement of the digested fragments of HERV-E or HERV-K.

**Figure 1:**
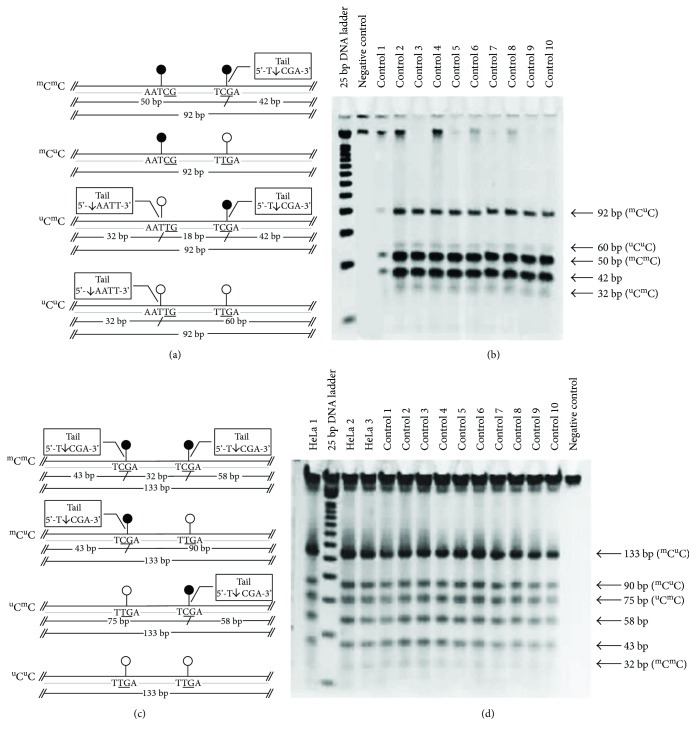
Combined-bisulfite restriction analysis (COBRA) for LINE-1 (a, b) and Alu (c, d) methylation patterns. The diagrams (a, c) demonstrate the following four patterns of methylated CpGs (from top to bottom): hypermethylation (^m^C^m^C), hypomethylation (^u^C^u^C), and two forms of partial methylation (^m^C^u^C and ^u^C^m^C). Polyacrylamide gel electrophoresis (b, d) shows the locations for the bands for each pattern of methylated CpGs. Quantitative DNA ladder was used to assess the size of the bands. The representative gels of cell line and normal control samples are shown. Water is used as a negative control.

**Figure 2:**
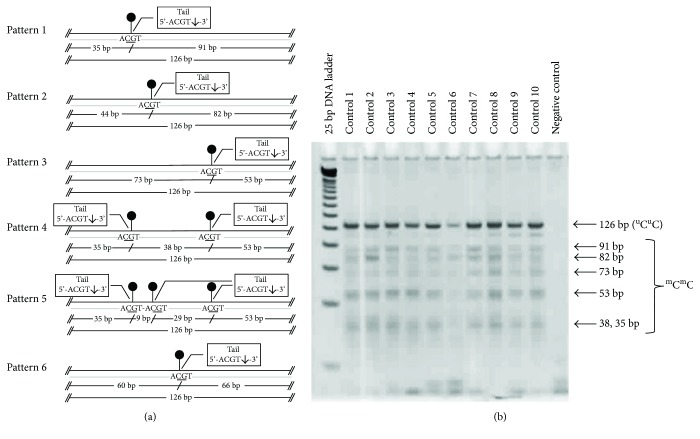
Combined-bisulfite restriction analysis (COBRA) for HERV-E methylation patterns. The diagrams (a) demonstrate six patterns of hypermethylation (^m^C^m^C). Polyacrylamide gel electrophoresis (b) shows the locations for the bands for each pattern of methylated CpGs. Quantitative DNA ladder was used to assess the size of the bands. The representative gels of cell line and normal control samples are shown. Water is used as a negative control.

**Figure 3:**
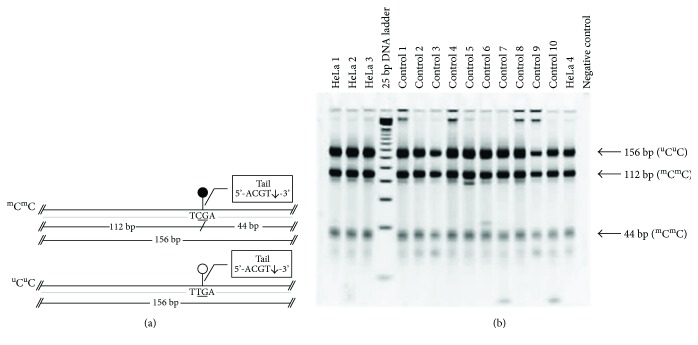
Combined-bisulfite restriction analysis (COBRA) for HERV-K methylation patterns. The diagrams (a) demonstrate the following two patterns of methylated CpGs (from top to bottom): hypermethylation (^m^C^m^C) and hypomethylation (^u^C^u^C). Polyacrylamide gel electrophoresis (b) shows the locations for the bands for each pattern of methylated CpGs. Quantitative DNA ladder was used to assess the size of the bands. The representative gels of cell line and normal control samples are shown. Water is used as a negative control.

Methylation bands for LINE-1, Alu, HERV-E, and HERV-K from normal control samples are shown in Figures 1–3, respectively.

### 2.6. Statistical Analyses

Wilcoxon rank-sum test (two-tailed) was used to compare the methylated levels between groups. *p* value < 0.05 was considered statistically significant. All analyses were performed utilizing Stata 13.0 (StataCorp, College Station, TX).

## 3. Results

### 3.1. Patient Characteristics

The demographic and clinical characteristics of the glaucoma patients and control subjects are shown in Table 1. All subjects were of Thai ethnicity. Most of the glaucoma patients had only trabeculectomy done. Combination of cataract surgery and trabeculectomy was performed in six, eight, and one patient from POAG, PACG, and secondary glaucoma groups, respectively. The diagnoses of primary eye condition in secondary neovascular glaucoma groups included proliferative diabetic retinopathy and central retinal vein occlusion (7 cases), uveitis (4 cases), trauma (3 cases), postcorneal surgery (1 case), and ICE syndrome (1 case). Three POAG cases and 3 PACG cases reported to have family history of glaucoma. However, it should be noted that only the results with clear patterns in the gel electrophoresis as shown in Figures 1–3 were included in the analysis. Therefore, the number of samples analyzed in Tables 2–5 are not always equal.

### 3.2. LINE-1 Methylation Analysis

**Figure 4:**
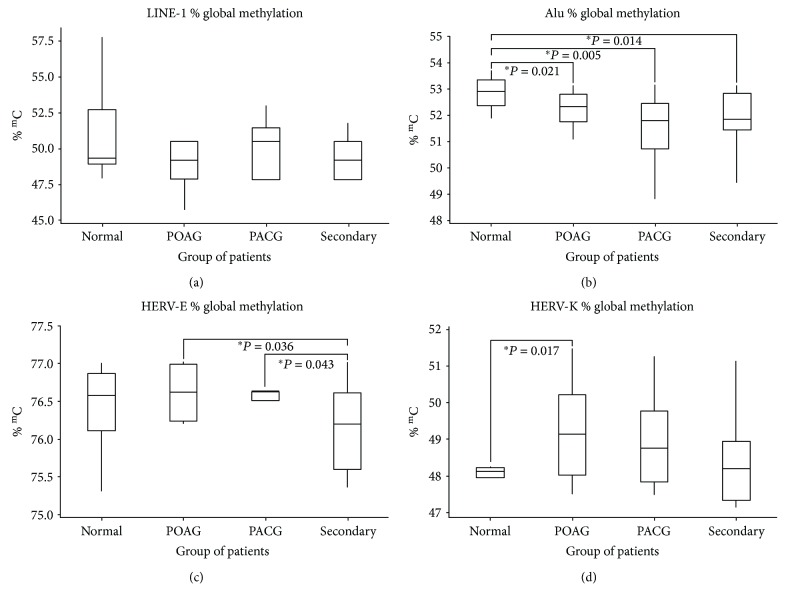
Box plots showing methylation percentage: (a) LINE-1 methylation analysis, (b) Alu methylation analysis, (c) HERV-E methylation analysis, and (d) HERV-K methylation analysis. POAG = primary open-angle glaucoma; PACG = primary angle-closure glaucoma; secondary = secondary glaucoma; % ^m^C = percentage of methylation. ^∗^ represents a significant difference at *p* < 0.05 by Wilcoxon rank-sum test.

The averages of the methylation percentages are shown in Table 2. There were no statistical significant differences in the overall methylation among the glaucoma patients and the normal controls or among the patients with different types of glaucoma (Figure 4(a)).

### 3.3. Alu Methylation Analysis

The average level of Alu methylation is shown in Table 3. The overall methylation was significantly lower in the tissues of the patients with all types of glaucoma compared to the controls: POAG (52.19%) compared to controls (52.83%), *p* = 0.021; PACG (51.50%) compared to controls, *p* = 0.005; and secondary glaucoma (51.95%) compared to controls, *p* = 0.014 (Figure 4(b)).

### 3.4. HERV-E Methylation Analysis

The level of HERV-E methylation is shown in Table 4. The overall methylation in the tissue was not statistically different among glaucoma patients compared to the controls. However, there was significantly lower methylation in patients from the secondary glaucoma group (75.95%) when compared to the POAG (76.61%) and PACG (76.50%) groups (*p* = 0.036 and *p* = 0.043, resp.) (Figure 4(c)).

### 3.5. HERV-K Methylation Analysis

The level of HERV-K methylation is shown in Table 5. The overall methylation in the tissue specimens was statistically higher for the POAG (49.22%) patients compared to the controls (48.09%) (*p* = 0.017) (Figure 4(d)).

## 4. Discussion

This study is the first epigenetic study conducted using ocular tissues from glaucoma patients. We demonstrated that the global methylation levels for Alu were significantly lower in POAG, PACG, and secondary glaucoma groups compared to the controls. Hypomethylation of HERV-E was also observed in the secondary glaucoma group. On the other hand, the methylation levels for HERV-K were significantly higher in the POAG group compared to controls.

The characteristic of high IOP found in most types of glaucoma is thought to be due to increased resistance in the trabecular outflow pathway. Gottanka et al. found that trabecular sheath plaques inside TM are significantly higher in patients with glaucomatous problems compared to patients with normal eyes [21]. The plaque materials were composed of fine fibrils and other components of the extracellular matrix that adhered to the sheaths of the TM fibers [22, 23]. Also, Sihota et al. [24] demonstrated the excessive fibrillary structure in the extracellular matrix in PACG patients, affecting the narrow TM beams. The area with such changes was away from the peripheral anterior synechiae. This finding may explain why after a successful angle widening in some PACG patients resulted in unsatisfactory reduction of the IOP.

There is evidence indicating that TGF-*β*2, a profibrotic cytokine, played a role in the changes of the extracellular matrix of the trabecular outflow pathway. TGF-*β*2 levels were documented to be higher in aqueous humor in nearly half of the patients with POAG [25]. In vitro, this cytokine can stimulate trabecular cells to increase the synthesis of various extracellular matrix components and tissue transglutaminase enzyme, which cross-links proteins to complexes not degradable by metalloproteinases [26]. The enzyme metalloproteinases are also inhibited by plasminogen activator inhibitor, which can be upregulated by TGF-*β*2 [23, 27]. Moreover, the eyes treated with TGF-*β*2 can result in substantially decreased outflow facility [28].

Aside from that, there is evidence suggesting the role of epigenetic regulation of the TGF-*β* pathway [29, 30]. The TGF-*β* signaling pathway has been shown to be suppressed by the methylation process. Treating cells with DNA methyltransferase (DNMT), the enzyme that is responsible for the transfer of methyl groups to the DNA can inhibit the TGF-*β* pathway activity [29]. In addition, the methylation also affects the level of thrombospondin-1 (TSP1), which is known to regulate the TGF-*β* pathway [31]. It is possible that changes in the methylation level may affect the TGF-*β* signaling pathways as well as the regulation of TSP1 level, subsequently increasing the production of extracellular matrix to form plaques in the TM.

Hypoxia has been shown to induce epigenetic changes in other fibrotic diseases as well as being implicated in the pathogenesis of glaucoma. Tezel and Wax [32] found that hypoxia-inducible factor 1*α* and its related hypoxia-induced proteins (i.e., vascular endothelial growth factor) level was increased in the retina and optic nerve head of glaucoma patients [33]. Decreased ocular blood flow [34], disturbed ocular autoregulation [35], and increased IOP or IOP fluctuation [33] may potentially cause intraocular hypoxia. Many reports suggested the role of hypoxia in inducing changes at the epigenetic level. Recently, McDonnell et al. [36] studied the hypoxic response in human lamina cribrosa cells and found that hypoxia significantly increased expression of DNMT and the levels of global DNA methylation when compared to the normoxic lamina cribrosa cells.

However, it is not clear why methylation change was observed only with certain subtypes of IRSs. There is a lot of evidence indicating that DNA methylation of the IRSs, particularly LINE-1 and Alu, are biomarkers for environmental exposures such as air pollution, metal exposure, and alcohol consumption [37]. The association between methylation and the exposures may vary between the markers for IRSs. Previous studies showed that there was an increase in the expression of Alu RNAs in response to cellular stress [38]. Alu RNA-induced cytotoxicity was also proposed to be implicated in age-related macular degeneration via inducing proinflammatory cytokine cascade [39]. On the other hand, an increased expression of HERV protein was documented in many studies that were associated with cytokines, hypoxia, microorganisms, steroid hormones, and even the environment [40–43].

In contrast to the Alu results in which the methylation change was found in all 3 glaucoma groups, the DNA hypermethylation for HERV-K was detected only in patients with POAG. This makes sense because POAG is known to have a very strong genetic predisposition [44, 45]. The HERV-K marker may be more specific to epigenetic modulation of genetic susceptible persons, whereas we speculate that the Alu marker is more susceptible to oxidative stress and hypoxic condition. However, this hypothesis needs to be confirmed by investigating how hypomethylation at Alu and hypermethylation at HERV-K may relate to the pathogenesis of glaucoma and to what extent these modulating factors contribute to such changes. Additional studies on the downstream effects of the expressions of Alu, HERV-K, and gene-specific methylation, are needed.

Our studies have some limitations. First, the tissues in this study were collected from patients who fulfilled the criteria for trabeculectomy. Consequently, the results cannot be generalized to the milder form of glaucoma and the methylation changes that may not be representative of the stage of the disease. Second, because it is unethical to obtain scleral/trabecular tissue from normal subjects, we collected the tissue from postmortem eyes. Hence, a detailed ocular examination by the authors was not possible. The inclusion criteria were thus based on having no history of glaucoma which might not totally exclude the existence of glaucomatous changes in this group. Third, the ages of the control samples were within a wide range with an overall lower average age compared to the glaucoma groups. The difference in age potentially affect the comparison of the methylation levels of Alu and HERV-K. A study from Jintaridth et al. found that both Alu and HERV-K methylation levels had an inverse correlation with age [46]. Our high methylation level of normal control compared to glaucoma groups in Alu could be a result of the age effect. Nevertheless, the hypermethylation in HERV-K despite the older age POAG subjects compared to the controls may represent the true difference of methylation level in this marker. It should be noted that the study from Jintaridth et al. was conducted in peripheral blood mononuclear cells and not the ocular tissue. Given that available control eyes were usually obtained from individuals who had an unnatural cause of death such as accident or trauma, they tended to be younger. This limited our ability to perform age-matching in our study. Lastly, since this is an exploratory study, each group contained small numbers of subjects which limited our ability to build multivariable models with various covariates due to low statistical power and overfitting concerns. Future studies with large numbers of subjects and age-matching design are warranted to confirm these potential associations with incorporation of potential confounders into a statistical model.

In conclusion, trabeculectomy sections from POAG, PACG, and secondary glaucoma patients had DNA hypomethylation at Alu, and DNA hypermethylation was detected at HERV-K for POAG patients. These methylation changes may lead to TM transformation and dysfunction. Our findings also suggest that epigenetic modulation may be a potential mechanism of glaucoma pathogenesis.

## Figures and Tables

**Table 1:** Demographic and clinical characteristics of the subjects.

	Normal control	POAG	PACG	Secondary glaucoma
*N*	10	16	12	16
*Age*				
Mean (SD)	40 (20.8)	69 (8.1)	65 (10.0)	53 (15.0)
*Gender, male*				
*N* (%)	5 (50.0)	11 (64.7)	2 (15.4)	9 (56.3)
*Laterality, right*				
*N* (%)	5 (50.0)	9 (52.9)	7 (58.3)	8 (50.0)
*Duration (month)*				
Median (IQR)	—	36.0 (24.0, 84.0)	12 (7.0, 132.0)	2.5 (1.75, 9.75)
*Vertical C : D ratio*				
Median (IQR)	—	0.9 (0.8, 0.9)	0.8 (0.8, 0.9)	0.8 (0.7, 0.9)
*IOP (mmHg)*				
Mean (SD)	—	20 (8.1)	21.6 (9.8)	33.3 (13.0)
*VA (decimal)*				
Median (IQR)	—	0.677 (0.200, 1.000)	0.500 (0.100, 0.700)	0.015 (0.001, 0.400)
*Number of medication*				
Median (IQR)	—	4.0 (3.75, 4.0)	4.0 (4.0, 4.3)	4.0 (4.0, 5.0)
*Visual field MD*				
Median (IQR)	—	−11.31 (−16.98, −9.43)	−10.62 (−14.85, −4.50)	NA
*Visual field PSD*				
Median (IQR)	—	7.75 (3.31, 8.21)	7.59 (1.98, 9.22)	NA

POAG = primary open-angle glaucoma; PACG = primary angle-closure glaucoma; C : D = optic cup to optic disc ratio; VA = visual acuity; MD = mean deviation; PSD = pattern standard deviation; NA = not applicable due to poor visual acuity.

**Table 2:** LINE-1 methylation levels in glaucoma eyes and control eyes.

	*N*	% ^m^C	% ^m^C^m^C	% ^m^C^u^C	% ^u^C^m^C	% ^u^C^u^C
Normal control	10	50.8810 ± 3.4356	18.2732 ± 2.5257	23.2248 ± 2.1280	24.1912 ± 0.4800	34.3107 ± 3.6554
POAG	16	49.8650 ± 3.0136	17.4822 ± 2.5099	23.7864 ± 1.8280	23.5895 ± 0.8576	35.1419 ± 2.5862
PACG	12	49.8900 ± 1.8009	17.9876 ± 2.2382	22.2586 ± 2.9002	23.5921 ± 0.4616	36.1617 ± 0.7060
Secondary glaucoma	14	49.3133 ± 1.5371	17.2674 ± 1.8776	23.0947 ± 2.4025	23.5456 ± 0.3893	36.0923 ± 0.6032

All values are presented as mean ± standard deviation. % ^m^C = percentage of LINE-1 methylation; % ^m^C^m^C = percentage of LINE-1 hypermethylated loci number; % ^m^C^u^C, % ^u^C^m^C = percentage of LINE-1 partially methylated loci; % ^u^C^u^C = percentage of LINE-1 hypomethylated loci number.

**Table 3:** Alu methylation levels in glaucoma eyes and control eyes.

	*N*	% ^m^C	% ^m^C^m^C	% ^m^C^u^C	% ^u^C^m^C	% ^u^C^u^C
Normal control	10	52.8278 ± 0.5926	25.1117 ± 1.2948	27.5755 ± 2.2979	27.8567 ± 2.4824	19.4560 ± 0.1800
POAG	16	52.1945 ± 0.6916	24.0020 ± 1.5875	27.5126 ± 2.2014	28.8725 ± 0.4600	19.6130 ± 0.3089
PACG	12	51.4995 ± 1.3884	22.6191 ± 2.7542	28.8866 ± 2.7807	28.8743 ± 0.3395	19.6201 ± 0.2302
Secondary glaucoma	16	51.9548 ± 0.9712	23.5236 ± 1.9268	27.9738 ± 2.0784	28.8885 ± 0.4241	19.6140 ± 0.2888

All values are presented as mean ± standard deviation. % ^m^C = percentage of Alu methylation; % ^m^C^m^C = percentage of Alu hypermethylated loci number; % ^m^C^u^C, % ^u^C^m^C = percentage of Alu partially methylated loci; % ^u^C^u^C = percentage of Alu hypomethylated loci number.

**Table 4:** HERV-E methylation levels in glaucoma eyes and control eyes.

	*N*	% ^m^C	% ^u^C
Normal control	10	76.4320 ± 0.5745	23.5680 ± 0.5745
POAG	12	76.6092 ± 0.3342	23.3908 ± 0.3342
PACG	10	76.4990 ± 0.4257	23.5010 ± 0.4257
Secondary glaucoma	13	75.9462 ± 1.0527	24.0538 ± 1.0527

All values are presented as mean ± standard deviation. % ^m^C = percentage of HERV-E methylation; % ^u^C = percentage of HERV-E nonmethylation.

**Table 5:** HERV-K methylation levels in glaucoma eyes and control eyes.

	*N*	% ^m^C	% ^u^C
Normal control	10	48.0928 ± 0.1242	51.9072 ± 0.1242
POAG	16	49.2227 ± 1.1984	50.7773 ± 1.1984
PACG	12	48.9118 ± 1.1630	51.0882 ± 1.1630
Secondary glaucoma	16	48.4693 ± 1.2119	51.5307 ± 1.2119

All values are presented as mean ± standard deviation. % ^m^C = percentage of HERV-K methylation; % ^u^C = percentage of HERV-K nonmethylation.
